# La pneumatose kystique intestinale secondaire à un ulcère peptique : à propos de trois cas

**DOI:** 10.11604/pamj.2015.20.150.3017

**Published:** 2015-02-17

**Authors:** Karim Ibn Majdoub Hassani, Issam Yazough, Said Ait Laalim, Imane Toughrai, Khalid Mazaz

**Affiliations:** 1 Faculté de Médecine et de Pharmacie de Fès, Université Sidi Mohammed Ben Abdellah, Département de Chirurgie, CHU Hassan II, Fès, Maroc

**Keywords:** Pneumatose kystique intestinale, pneumopéritoine, ulcère peptique, intestinal cystic pneumatosis, pneumoperitoneum, peptic ulcer

## Abstract

La pneumatose kystique intestinale (PNK) est une pathologie rare qui se caractérise par la présence de kystes gazeux dans la paroi intestinale. Elle est asymptomatique ou pauci symptomatique, et le plus souvent découverte lors d'un examen d'imagerie ou d'endoscopie. Nous rapportons une série de trois cas de pneumatose kystique secondaire à un ulcère peptique. A travers ce travail nous essayons de mettre la lumière sur les différents aspects cliniques, radiologiques et thérapeutiques de cette pathologie.

## Introduction

Le pneumopéritoine, se défini par la présence d'air dans la cavité péritonéale, et signe généralement la perforation d'un viscère creux intra-abdominal justifiant ainsi une exploration chirurgicale en urgence. L'origine peut être abdominale, thoracique ou gynécologique. Parmi les causes abdominales, la PNK est une étiologie classique, les étiologies de cette dernières restent multiples et l'ulcère peptique compte parmi ces étiologies. Le diagnostic n'est pas toujours évident, et seul un bilan radiologique et endoscopique correct est garant d'une prise en charge thérapeutique adéquate évitant pas mal de fois des laparotomies inutiles en urgences.

## Patient et observation

### Observation 1

**Figure 1:**
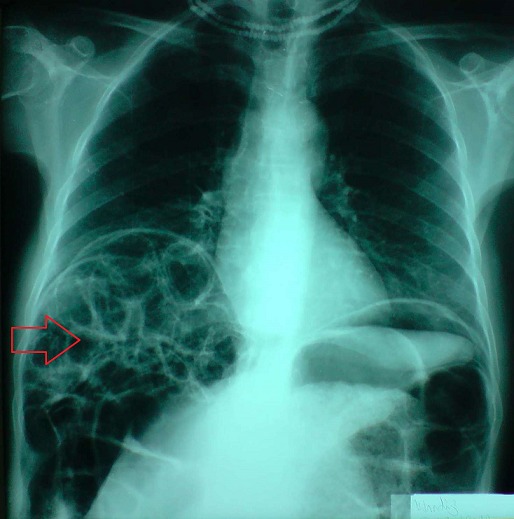
Cliché d'ASP montrant un pneumopéritoine bilatéral avec signe de Moreau Chilaïditi correspondant à l'interposition de multiples grappes de bulles entre le foie et la coupole diaphragmatique droite (flèche rouge)

**Figure 2:**
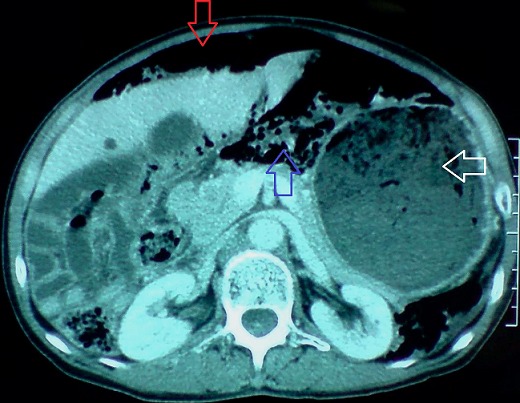
Coupe axiale de TDM abdominal montrant un important estomac de stase flèche blanche) associé à un pneumopéritoine (flèche rouge) et à une PNK diffuse (flèche bleue)

**Figure 3:**
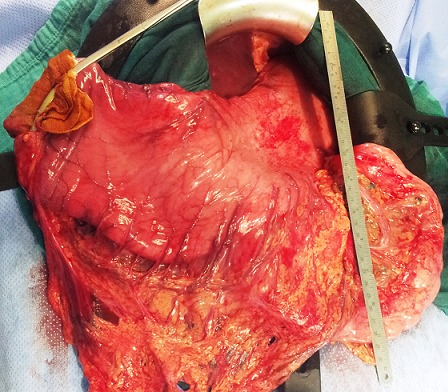
Vue per opératoire montrant l’énorme estomac de stase et l'absence de pneumatose kystique

Patiente âgée de 59 ans, suivie en consultation de gastroentérologie pour une sténose bulbaire avec gastrite ulcérée sous traitement médicamenteux à base d'IPP depuis 6 mois, admise aux urgences pour des douleurs épigastriques intenses sans vomissement ni troubles de transit. L'examen clinique retrouve une patiente apyrétique, avec à l'examen abdominal une sensibilité épigastrique et une distension abdominale diffuse. Le bilan biologique est sans anomalie en dehors d'une légère hypokaliémie à 3,3. Les clichés d'abdomen sans préparation montrent un pneumopéritoine bilatéral (Figure 1) sans niveau hydro-aérique. Nous avons donc compléter le bilan radiologique par une échographie abdominale qui a montré une ascite de faible abondance. La TDM abdominale (Figure 2) a montré un important estomac de stase associé à un pneumopéritoine, à une PNK diffuse et à un épanchement liquidien péritonéal de faible abondance localisé au pelvis. Devant l'absence de signe d'irritation péritonéale et de syndrome infectieux clinico-biologique franc, la malade a été transféré au service de chirurgie et a été programmé pour une laparotomie exploratrice quelques jours plu tard. L'exploration chirurgicale a montré la disparition totale de la PNK avec un énorme estomac de stase atone (Figure 3) d'où la décision de réalisé une bivagtomie tronculaire avec antrectomie et gatsro-entro-anastomose. Les suites opératoires étaient simples. Actuellement, à plus d'un an de recul la malade est asymptomatique.

### Observation 2

**Figure 4:**
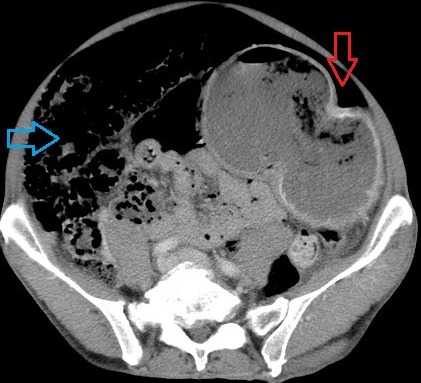
Coupe axiale de TDM abdominale objectivant une PNK intestinale (flèche bleue) et un pneumopéritoine de moyenne abondance (flèche rouge)

Patient âgé de 50ans, ayant comme antécédents la notion d’épigastralgies à répétition avec des vomissements post prandiaux tardif depuis plusieurs années rebelles aux traitements symptomatiques admis aux urgences pour des douleurs avec distension abdominale depuis plusieurs jours évoluant dans un contexte d'altération de l’état général. L'examen clinique retrouve un patient apyrétique légèrement tachycarde à 95bat/min, avec à l'examen abdominal la Présence d'un tympanisme diffus sans défense ni contracture abdominale. L'ASP montre un pneumopéritoine bilatéral. Le bilan biologique ne montre pas d'anomalie. Une TDM TAP a objectivé une PNK intestinale compliquée d′un pneumopéritoine de moyenne abondance (Figure 4). Nous avons compléter le bilan par une FOGD qui a montré la présence d'une sténose bulbaire difficile à franchir avec estomac de stase. Le patient a été mis sous traitement IPP pendant 8 semaines sans véritable amélioration avec à la FOGD de contrôle la persistance d'une sténose bulbaire infranchissable. Le malade fut opéré par la suite et a bénéficié d'une bi vagotomie tronculaire avec gastro-entero-anastomose, à noté que l'exploration chirurgicale ne retrouvait pas de PNK. Les suites opératoires étaient sans particularités.

### Observation 3

**Figure 5:**
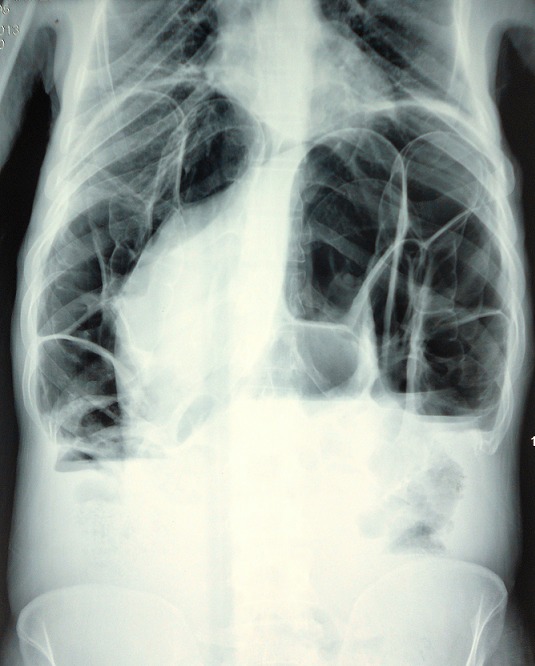
L'ASP debout montre un pneumopéritoine important avec aérocolie et des niveau hydroaeriques

**Figure 6:**
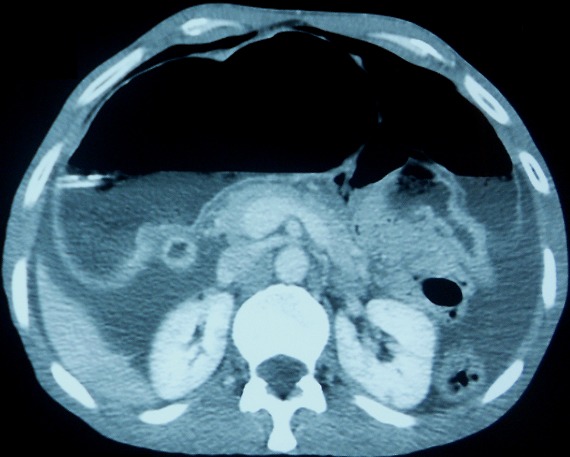
Coupe axiale de TDM abdominal montrant un estomac de stase et un important hydro-pneumopéritoine

**Figure 7:**
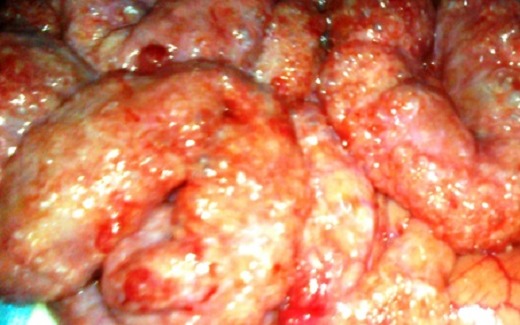
Vue per opératoire montrant une PNK intestinale diffuse

Patient âgé de 50 ans, suivi au service de gastroentérologie depuis 3ans pour une sténose peptique du pylore sous traitement IPP, Qui présente depuis 3 mois des douleurs abdominales à type de pesanteur sans signes accompagnateurs évoluant dans un contexte d′apyrexie et d′AEG. Le malade a été admis aux urgences dans un tableau de distension abdominale avec déshydratation et altération de l’état général. L'examen général retrouve un patient déshydraté en mauvais état général apyrétique avec à l'examen abdominal un abdomen distendu sensible dans son ensemble. Le bilan biologique réalisé montre une numération formule sanguine correcte en dehors d'une hyperleucocytose à 12300élements /mm3 et une légère insuffisance rénale avec l'urèe à 0,76 et la créatinine à 21. L'ASP debout montre un pneumoperitoinne important avec aerocolie et niveau hydroaerique (Figure 5). Une TDM abdominale a été réalisée et est revenue en faveur d′une sténose antro-pylorique avec estomac de stase et un important hydro-pneumopéritoine (Figure 6). Après une préparation Le patient a été admis au bloc opératoire pour exploration chirurgicale qui a retrouvé un épanchement intra péritonéal fait d′environ 1litre d′ascite prélevé puis aspiré, avec présence de lésion de PNK au niveau de l'intestin grêle (Figure 7) et un estomac de stase atone arrivant jusqu′au pelvis. Une jejunostomie d'alimentation a été réalisée. Le malade a été réopéré deux semaines plu tard avec réalisation d'une bi vagotomie tronculaire, d'une antrectomie avec gastro-entero-anastomose, et là encore on note la disparition complète de la PNK. Les suites opératoires étaient sans particularités.

## Discussion

La pneumatose kystique intestinale (PKI) est une maladie bénigne rare, décrite pour la première fois par le français Duvernoy à la fin du XVIIe siècle, caractérisée par la présence de kystes à contenu gazeux (pneumokystes), habituellement multiples, de taille et de distribution variable, Ces lésions peuvent siéger de l′œsophage au rectum, mais prédominent au niveau du grêle et du côlon [1]. Ils sont généralement sous-muqueux dans le côlon, prenant l′aspect de nodules polypoïdes sessiles, ils sont plus souvent sous-séreux dans l′intestin grêle, revêtant la forme de bulles en grappes de raisin, et se situent alors surtout sur le bord mésentérique [2]. La paroi des kystes est parfois très fine et peut être rompue, soit spontanément, soit après une biopsie endoscopique provoquant un véritable pneumopéritoine [3, 4]. De nombreuses causes ont été décrites [5]: maladies inflammatoires intestinales, maladies obstructives pulmonaires ou gastrointestinales, hémopathies malignes, collagénoses, ulcère peptique ce qui est le cas de nos trois observations. L′endoscopie digestive traumatique, les greffes d′organes, le traitement immunosuppresseur et la corticothérapie prolongée sont aussi d′autres causes de la PNK. Les mécanismes physiopathologiques évoqués sont multiples et restent mal connue (rupture mécanique de la muqueuse digestive par hyperpression intraluminale, processus inflammatoire, infectieux ou médicamenteux fragilisant cette muqueuse). La PKI est généralement paucisymptomatique. L′âge de découverte se situe entre 40 et 50 ans avec un sex-ratio de 1:1 [6].

Dans la plupart des cas, la symptomatologie digestive est aspécifique, faite de douleurs abdominales vagues, d'intensité variable, diffuses ou localisées et accompagnées de troubles du transit. Certaines complications rares (3% des cas) liées au volume kystique ont été décrites: volvulus, invagination, perforation, hémorragie [7]. C'est pour ces raisons que le diagnostic repose essentiellement sur les examens complémentaires. Ainsi, le diagnostic peut être évoqué dès l'ASP devant des images aériques arrondies accolées en grappes de raisin ou en chapelet, bordant la paroi du tube digestif. Deux signes indirects sont importants à chercher [8]: le signe de Moreau Chilaïditi qui correspond à l'interposition de multiples grappes de bulles entre le foie et la coupole diaphragmatique droite et un éventuel pneumopéritoine témoignant de la rupture d'un kyste sous-séreux. Ce pneumopéritoine, présent dans 15% des atteintes du grêle et 3% des atteintes coliques, est souvent source d'erreur et d'interventions chirurgicales non justifiées [9]. La tomodensitométrie avec opacification intestinale possède une bonne précision diagnostique [10]. Elle révèle des images de densité gazeuse dans la paroi digestive, mieux visibles en section transversale et en fenêtre pulmonaire [8, 11]. L'association à un pneumopéritoine asymptomatique est quasi pathognomonique [2]. On a décrit un aspect échographique associant un amincissement de la paroi intestinale et des échos avec ombre acoustique, réalisant le «signe de l'aurore» [12]. Il existe un critère diagnostique important qui est l'absence d'aéroportie (à la différence des gangrènes intestinales) à la tomodensitométrie ou l’échographie [13]. En endoscopie, les kystes correspondent à de larges polypes sessiles hémisphériques, recouverts d'une muqueuse pâle et transparente, parfois ulcérée. Typiquement, on obtient l'affaissement du kyste à la ponction ou la biopsie avec un bruit d’éclatement [14]. L’évolution spontanée de la PKI qu'elle soit d'origine ulcéreuse ou autre est favorable dans la majorité des cas avec une régression totale des kystes. Certaines formes peuvent avoir une évolution chronique avec des périodes de rémission et de rechute [15]. Le traitement de la PKI est médical et empirique. Il associe une antibiothérapie per os dirigée contre les anaérobies [16] (le métronidazole est le plus souvent prescrit), une oxygénothérapie hyperbare ou un régime sans résidus pauvre en hydrates de carbone [17]. Les modalités et la durée de ce traitement restent discutées. Le taux de récidive est important (50% des cas) et reste lié aux affections associées d'où l'intérêt d'un traitement étiologique [18].

Le traitement chirurgical est indiqué en cas de complications et en cas de symptomatologie rebelle au traitement médical [19, 20]. Il consiste à réséquer le segment intestinal atteint par laparotomie ou encore mieux par laparoscopie. Ce dernier abord est préféré du fait de la bénignité de la pathologie et des conditions locales favorables (absence d'inflammation, absence d'adhérence imputable à cette pathologie).

## Conclusion

Le diagnostic de pneumatose kystique intestinale doit être évoqué dés l'imagerie. La discordance entre les signes radiologiques et la symptomatologie doit attirer l'attention. Le traitement est médicale lié aux hypothèses physiologiques. La PNK secondaire à un ulcère peptique reste rare, son évolution est généralement favorable et son traitement repose essentiellement sur celui de la sténose ulcéreuse, il peut être médical ou chirurgical. La connaissance de cette pathologie rare permet d’éviter une laparotomie exploratrice inutile.
